# Promotion of trophoblast invasion by lncRNA MVIH through inducing Jun‐B

**DOI:** 10.1111/jcmm.13400

**Published:** 2017-10-30

**Authors:** Yanfen Zou, Qian Li, Yetao Xu, Xiang Yu, Qing Zuo, Shiyun Huang, Yongli Chu, Ziyan Jiang, Lizhou Sun

**Affiliations:** ^1^ Department of Obstetrics and Gynecology The Affiliated Yantai Yuhuangding Hospital of Qingdao University Yantai Shandong Province China; ^2^ Department of Obstetrics and Gynecology The First Affiliated Hospital of Nanjing Medical University Nanjing JiangSu Province China; ^3^ Department of General Surgery The Affiliated Yantai Yuhuangding Hospital of Qingdao University Yantai Shandong Province China

**Keywords:** long non‐coding RNAs, MVIH, Preeclampsia, Jun‐B

## Abstract

Preeclampsia (PE), a pregnancy‐specific disorder, is associated with impaired uterine spiral artery remodelling, which is related to the dysfunction of trophoblast cells. Lately, mounting evidence has indicated that aberrant expression of long non‐coding RNAs (lncRNAs) is associated with various human diseases. The lncRNA *MVIH* transcript has been shown to decrease the severity of several diseases. However, the biological function of MVIH, which is down‐regulated in placental tissues in PE, has not yet been clarified. Here, we report that *MVIH* may act as a vital factor in the pathogenesis of PE. In this study, functional analysis revealed that the silencing of *MVIH* expression *via* transfection with small interfering RNA (siRNAs) inhibited cell growth, migration, invasion, and angiogenesis in various trophoblast cell lines, and stimulation with *MVIH* could promote these functions. Mass spectrometry analysis revealed that MVIH could modulate Jun‐B protein expression, which has been reported to potentially regulate cell growth and angiogenesis. Further cotransfection assays were performed, revealing that *MVIH* and *Jun‐B* have a synergistic effect on the regulation of angiogenesis and cell proliferation. Taking these findings together, *MVIH* could be associated with PE and may be a candidate biomarker for its diagnosis and treatment.

## Introduction

Preeclampsia (PE) is a pregnancy‐specific disease characterized by hypertension and proteinuria 1. It remains a leading cause of maternal mortality and morbidity worldwide 2. Shallow extravillous trophoblast (EVT) invasion and impaired spiral artery remodelling are thought to be involved in the pathogenesis of PE 3. Trophoblast dysfunctions, such as inhibited proliferation 4, anti‐angiogenesis 5 and decreased migration and invasion 6, make major contributions to the failure of spiral artery remodelling. Hence, to deepen our understanding of this disease, it is important to evaluate the molecules underlying the biological functions of trophoblasts.

With rapid innovations in whole‐genome sequencing techniques, the significance of non‐coding RNAs (ncRNAs) for a range of biological processes has been determined, particularly that of long non‐coding RNAs (lncRNAs) 7, 8, 9. lncRNAs are a newly identified class of non‐coding RNAs that are more than 200 nt in length with limited or no protein‐coding capacity. The human genome contains approximately 7000–23,000 lncRNAs 10. Multiple lines of evidence have revealed the contribution of lncRNAs to a wide spectrum of biological processes, including the induction of disease, X‐chromosome inactivation in embryonic development, chromatin modification in substance metabolism and the dysfunction of cells in tumorigenesis 11, 12, 13, 14. Moreover, accumulating evidence indicates that deregulated lncRNAs are closely related to the pathogenesis of PE 15, 16.

Our previous studies also revealed a panel of lncRNAs that might serve as potential biomarkers for predicting PE 17, 18. Among these, abnormal expression of *SPRY4‐IT1*, a highly conserved lncRNA that is localized in the nucleus, might be predictive of trophoblast metastasis and apoptosis in PE 17. Findings also suggested that down‐regulated lncRNA *MEG3* in PE might account for the promotion of trophoblast apoptosis and suppression of trophoblast invasion 18. Moreover, after screening for lncRNAs that exhibited differential expression between PE placentas and normal cases, another lncRNA, *MVIH* (microvascular invasion in hepatocellular carcinoma), was examined.

MVIH is an lncRNA that was only recently identified; it was shown to have the potential to modulate angiogenesis and cell invasion 19. The significantly lower level of *MVIH* in PE placentas identified in this previous work implied its regulatory role in this disease. However, the mechanisms underlying this remained to be elucidated. Against this background, in this study using *in vitro* assays, we explored the regulatory pathway of *MVIH* in trophoblast invasion and tube formation potential in PE. Our results provide novel insights into the biological functions of *MVIH*, which may be a latent biomarker for PE diagnosis and gene therapy.

## Materials and methods

### Tissue samples and clinical data collection

Thirty paired placental tissues from women with PE and controls were obtained, after which all obtained tissue samples were instantly snap‐frozen in liquid nitrogen and stored at −84°C before RNA extraction. The detailed clinic characteristics of the patients are recorded in Table 1. Our study was approved by the Ethics Committee of the First Affiliated Hospital of Nanjing Medical University, China. Written informed consent was obtained from all of the subjects in this study.

**Table 1:** Clinical characteristics of normal and preeclamptic pregnancies

Variable	PE (*n* = 30)	*N* (*n* = 30)	*P* value Control *versus* PE
Maternal age	30.2 ± 5.7	30.6 ± 3.5	>0.05
Proteinuria (g/day)	> 0.3	<0.3	<0.01
Gestational age (week)	36.5 ± 3.7	39.1 ± 1.2	>0.05
Systolic blood pressure, mm Hg	169 ± 20.1	112 ± 6.8	< 0.01
Diastolic blood pressure, mm Hg	115 ± 12.8	77 ± 7.1	<0.01
Body weight of infant (g)	2582 ± 740	3322 ± 413	<0.05
CRP (C‐reaction proteins)	8.1 ± 3.1	5.9 ± 2.9	>0.05
Maternal smoking(number)	2	1	>0.05

All results are presented as mean ± S.D. S.D., standard deviation.

Obtained by one‐way analysis of variance using SPSS 18.0 software (SPSS Inc, Chicago, IL, USA).

### Cell culture

HTR‐8/SVneo, an immortalized first‐trimester EVT cell line 20 that originated from a short‐lived primary EVT cell line, was generously provided by Dr. Charles Graham, Queen's University, Canada. With the behaviour of trophoblast cells, many studies 21, 22 have used this cell line to simulate trophoblast cells in pregnancy.

Human choriocarcinoma cells (JEG‐3) and human umbilical vein endothelial cells (HUVEC) were obtained from the Chinese Academy of Sciences Committee (Shanghai, China). HTR‐8/SVneo, JEG‐3 and HUVEC‐C were incubated in RPMI 1640 (Gibco, Nanjing, China), DMEM (Gibco, Nanjing, China), and ECM (Gibco, Invitrogen, Carlsbad, CA, USA), respectively, which had been supplemented with 5% FBS (Gibco, Invitrogen), 100 U/ml penicillin and 100 mg/ml streptomycin in humidified air at 37°C with 5% CO_2_.

### RNA preparation and qRT‐PCR

Total RNA was isolated using TRIzol reagent (Invitrogen, Grand Island, NY, USA), and qRT‐PCR analyses were performed in accordance with the manufacturer's manual (Takara, Dalian, China). A reverse transcription kit (Takara) was used for the synthesis of cDNA. The amplification of cDNA was performed using Power SYBR Green (Takara) in a reaction mix for qRT‐PCR with a total volume of 20 μl. In accordance with the manual, the reverse transcription was implemented at 37°C for 15 min. and 85°C for 5 sec. The qRT‐PCR results were analysed and expressed relative to threshold cycle (CT) values, and then converted to fold changes. The related primer sequences and siRNAs are presented in Table S1.

### Transfection of cell lines

HTR‐8/SVneo, JEG3 and HUVEC‐C cell lines were plated in six‐well plates and then transfected with corresponding siRNAs (10 μl) or plasmid vectors (4 μg) using Lipofectamine 2000 (Invitrogen) and plasmid vectors, respectively. pIRES2‐EGFP and pIRES2‐*MVIH* were extracted using DNA Midiprep kit (Qiagen, Hilden, Germany). At 48 hrs after treatment, trophoblast cells were harvested for further experiments, as exemplified by qRT‐PCR and Western blotting.

### Cell proliferation assays

Cell viability was determined using the MTT assay (Cell Proliferation Reagent Kit I; Roche Applied Science, Penzberg, Germany). The cell lines were transfected with si‐*MVIH* or pIRES2‐MVIH (3000 cells/well) and were plated in 96‐well plates with five duplicates. Cell viability was tested every 24 hrs, in accordance with the instructions. The absorbance was detected at 490 nm with an ELx‐800 University Microplate Reader (BioTek, Winooski, VT, USA).

### Flow cytometry

Flow cytometry was performed to determine and quantify the phases of cells within the cell cycle. Cells for cell cycle analysis were stained with propidium oxide using the Cycle TEST PLUS DNA Reagent Kit (BD Biosciences, Franklin Lakes, NJ, USA), in accordance with the manufacturer's manual, and analysed by FACScan. The proportions of cells in the G_0_–G_1_, S and G_2_–M phases were determined and compared.

### Transwell assays

Cell migration and invasion abilities were analysed by Transwell assays. A total of 3 × 10^4^ to 5 × 10^4^ cells were plated on the top of a membrane precoated with Matrigel (BD Biosciences; without Matrigel for cell migration assays). Upon incubation for 24–48 hrs, cells inside the upper chamber were removed with cotton swabs, while cells on the lower membrane surface were fixed with methanol and then stained with 0.5% Crystal violet solution. Five randomly selected fields were counted in each well.

### Network formation assay

Previous studies revealed that HTR‐8/SVneo and HUVEC‐C cells showed endothelial cell‐like behaviour regarding their ability to form tube‐like networks when grown on a Matrigel. The network formation assay was thus performed here to determine the ability of cells to undergo tube formation, as previously reported by Zou *et al*. 17. A total of 2.5 × 10^**4**^ cells treated with si‐MVIH or pIRES2‐MVIH were cultured in the upper layer of the chamber previously coated with Matrigel. After 6 hrs, images from five areas of each 96‐well plate were captured using a fluorescent microscope.

### Mass spectrometry analysis

To identify the relevant proteins targeted by MVIH, the bands that were particularly intense in the MVIH sample were selected for further analysis. These bands were excised for mass spectrometry analysis, which was performed by Shenzhen Genomics Institute (Shenzhen, China). Those proteins sequenced with at least two peptides were considered as a reliable identification. The detailed mass spectrometry data and information on protein identification are provided in Table S2.

### Statistical analysis

All statistical analyses were performed using SPSS 18.0 software (Armonk, NY, USA). The data are presented as the mean ± S.D. Statistical significance was assigned at *P *<* *0.05 (*) or *P *<* *0.01 (**).

## Results

### *MVIH* expression is down‐regulated in placental tissues of PE

**Figure 1:**
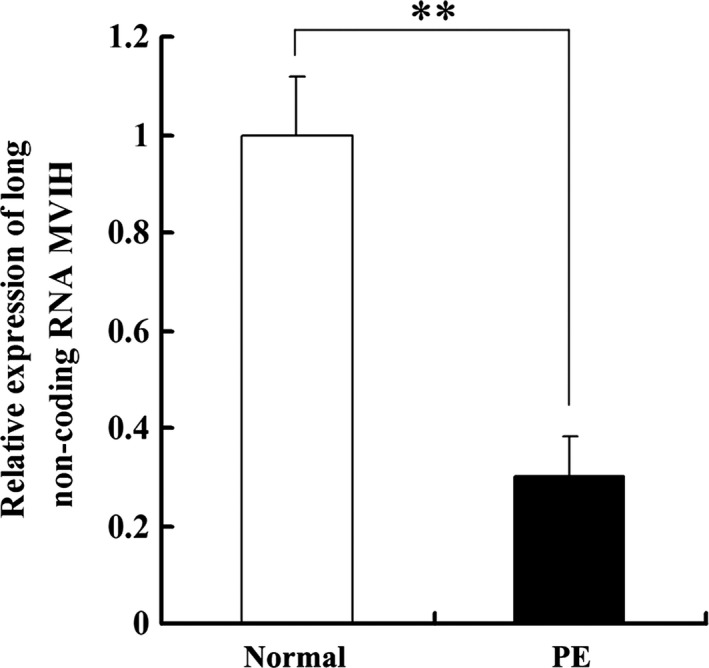
LncRNA *MVIH* expression is decreased in PE placentas. The relative expression of lncRNA MVIH was assessed by qPCR using SYBR green and normalized to GAPDH. The levels of MVIH were lower in preeclamptic placentas (*n *=* *30) than that in normal placentas (**A**) (*n *=* *30). (Values are mean ± SD; **: *P* < 0.01)

To explore the biological function of *MVIH* in placental tissue samples in cases of PE, we first determined the expression levels of *MVIH* in 30 paired placental tissues from PE and normal pregnancies by performing quantitative PCR (qPCR). The levels of *MVIH* transcript were markedly down‐regulated in PE placental tissues compared with their levels in placental tissue samples from normal pregnancies (*P *<* *0.01; Fig. 1). The detailed clinical characteristics, including systolic blood pressure, diastolic blood pressure, proteinuria and foetal birth weight, are presented in Table 1. The results indicate that MVIH might be a vital predictive biomarker for PE patients.

### Up‐regulation and down‐regulation of *MVIH* in trophoblast cell lines

**Figure 2:**
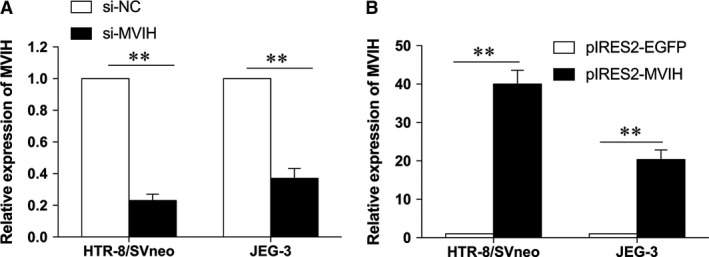
The transfection efficiency of si‐*MVIH* and pIRES2‐*MVIH*. The transfection efficiency was assessed by qPCR. The relative expression of MVIH decreased 80% and 70% after transfection of si‐MVIH in HTR‐8/SVneo and JEG‐3 cell lines, respectively (**A**) and the relative expression of MVIH was increased 40‐fold and 22‐fold after transfection of pIRES2‐MVIH in HTR‐8/SVneo and JEG‐3 cell lines, respectively (**B**). (Values are mean ± SD; **: *P* < 0.01)

To estimate the functional role of *MVIH* in biological behaviour, a small interfering RNAs (siRNAs) were first designed to silence *MVIH*. The results of qPCR analysis revealed that *MVIH* was significantly silenced by siRNAs in diverse human trophoblast cell lines, HTR‐8/SVneo and JEG‐3 (Fig. 2A). Meanwhile, we induced the ectopic overexpression of *MVIH* by transfecting trophoblast cell lines with the pIRES2‐*MVIH* expression vector (Fig. 2B). The qPCR assay was used to determine the efficiency of overexpression, which was 38‐fold and 22‐fold compared with the level in the negative control in HTR‐8/SVneo and JEG‐3 cells, respectively.

### Down‐regulation of *MVIH* inhibits trophoblast cell proliferation *in vitro*

**Figure 3:**
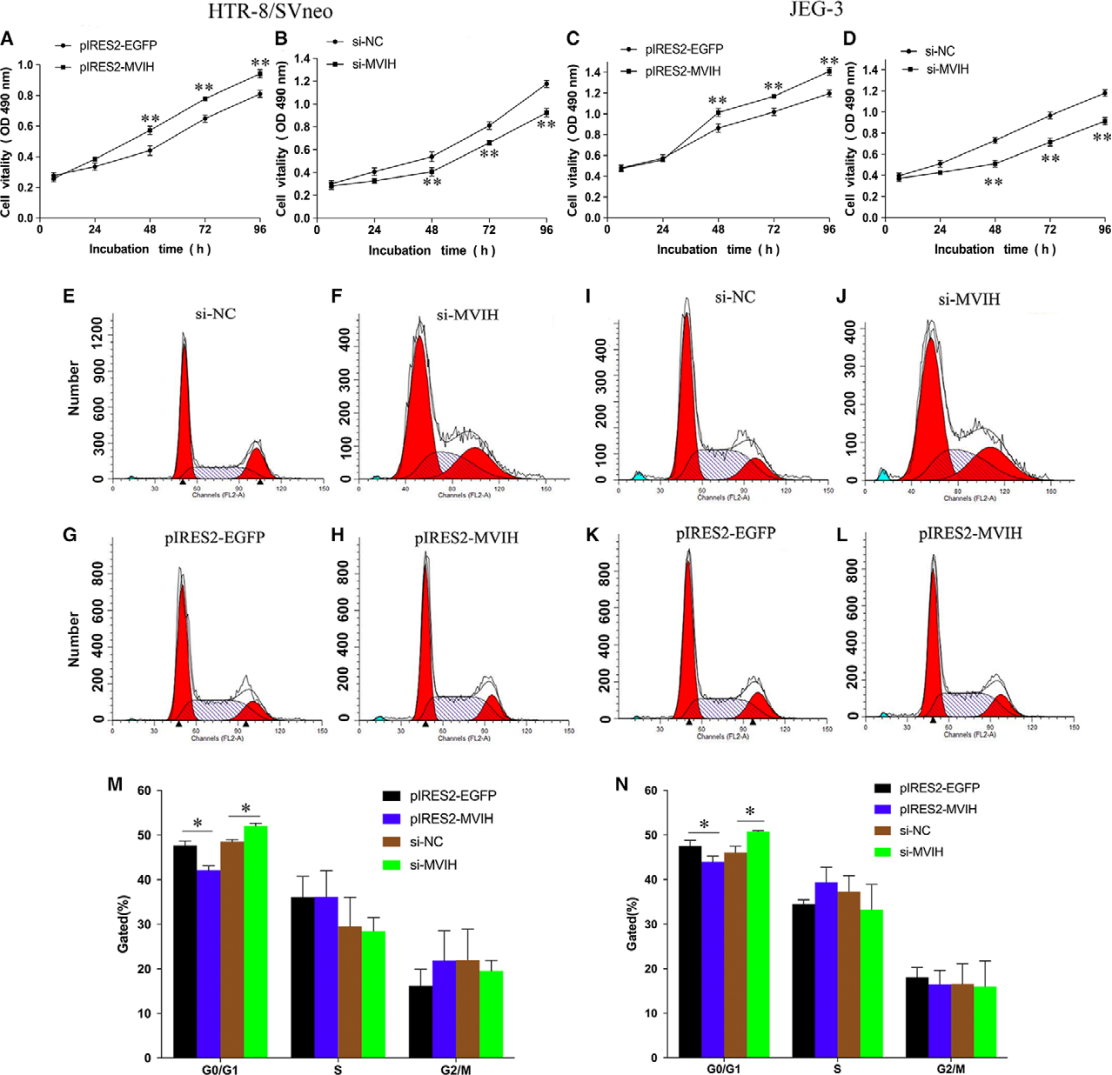
Down‐regulation of *MVIH* inhibited trophoblast cell proliferation *in vitro*. MTT assays were used to determine the viability of si‐MVIH‐transfected or pIRES2‐*MVIH*‐transfected Trophoblast Cells (**A–D**). Experiments were performed in triplicate. Cell cycle analyses by Flow cytometry in HTR‐8/SVneo cells and JEG‐3 cell lines. Representative fluorescence activated cell sorting images and statistics based were presented (**E–N**). (Values are mean ± SD; *: *P* < 0.05, **: *P* < 0.01)

At 48 hrs after transfection, we performed MTT assays and the resulting data revealed that the knockdown of *MVIH* expression considerably inhibited cell growth in HTR‐8/SVneo and JEG‐3 cells compared with that in the controls (Fig. 3A and C). Consistent with this, the stimulation of *MVIH* expression facilitated cell proliferation in HTR‐8/SVneo and JEG‐3 cells (Fig. 3B and D).

For further examination of whether cell cycle regulation was affected by the knockdown or overexpression of *MVIH*, we then performed flow cytometry to examine cell cycle progression. The obtained results implied that siRNA‐*MVIH* treatment or overexpression of *MVIH* affected the proportion of cells in the S phase and in the G_0_–G_1_ phase compared with those transfected with negative control (si‐NC) in HTR‐8/SVneo (Fig. 3E–I). Similarly, the same treatment resulted in the same trend in the JEG‐3 cell line (Fig. 3J–N).

### Effects of *MVIH* on the migration and invasion of trophoblast cell lines

**Figure 4:**
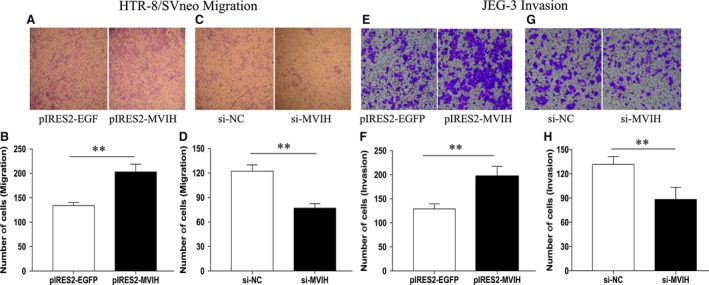
Effects of *MVIH* on the migration and invasion of two trophoblasts cell lines. The migration and invasion capacity of the cells transfected with si‐*MVIH* were significantly lower than that of the negative control and higher in the cells overexpressing pIRES2‐*MVIH*, as determined by Transwell assays (Values are mean±S.D.; **P <* *0.01) (**A–H**).

The migration and invasion of trophoblast cells are crucial for the progression of diverse diseases, particularly PE 17. Therefore, we investigated the effects of *MVIH* on the invasion and migration of trophoblast cells by performing Transwell assays. As shown in Figure 4A and B, the overexpression of *MVIH* stimulated cell migration. Conversely, the down‐regulation of *MVIH* inhibited the migration ability of HTR‐8/SVneo, with a considerable decline in the number of migratory cells (Fig. 4C and D). As shown in Figure 4E–H, silencing of *MVIH* impeded the invasion of JEG‐3, while *MVIH* overexpression had the opposite effect. These results indicate that *MVIH* can promote the migration and invasion of trophoblast cells.

### Effects of *MVIH* on network formation ability *in vitro*

**Figure 5:**
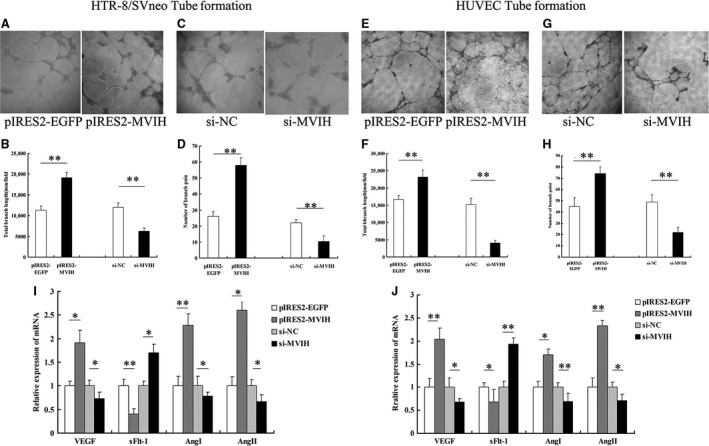
Effects of *MVIH* on network formation ability *in vitro*. Cells transfected with siRNAs targeting *MVIH* showed an increase in node numbers as compared to the negative control while cells treated with plasmid overexpressing pIRES2‐*MVIH* presented a decrease in node numbers as compared to that of the pIRES2‐EGFP (**A–H**). The expression of relevant vascular endothelial growth factor (VEGF) by qPCR after transfecting with effective siRNAs and pIRES2‐*MVIH*, respectively. (**I** and **J**) (Values are mean±S.E.M.; *P <* *0.05; **P <* *0.01).

Previous studies indicated that impaired spiral artery remodelling is involved in the pathogenesis of PE and resulted in placental ischaemia, hypoxia, and the release of a variety of placental factors into the maternal blood circulation 3, 23, which promoted activation of the systemic inflammatory response and vascular endothelial injury, in turn potentially leading to a systemic reaction. In this study, we investigated the influence of *MVIH* on the network formation ability of HUVEC and HTR‐8/SVneo cells, and shed light on the involvement of *MVIH* in the pathogenesis of PE. As shown in Figure 5A, B, E and F, the overexpression of *MVIH* boosted the network formation ability of HTR‐8/SVneo and HUVEC‐C cells. Conversely, the knockdown of *MVIH* inhibited the network formation ability and considerably decreased the number of capillary‐like networks, as revealed by the network formation assay *in vitro* (Fig. 5C, D, G, and H).

**Figure 6:**
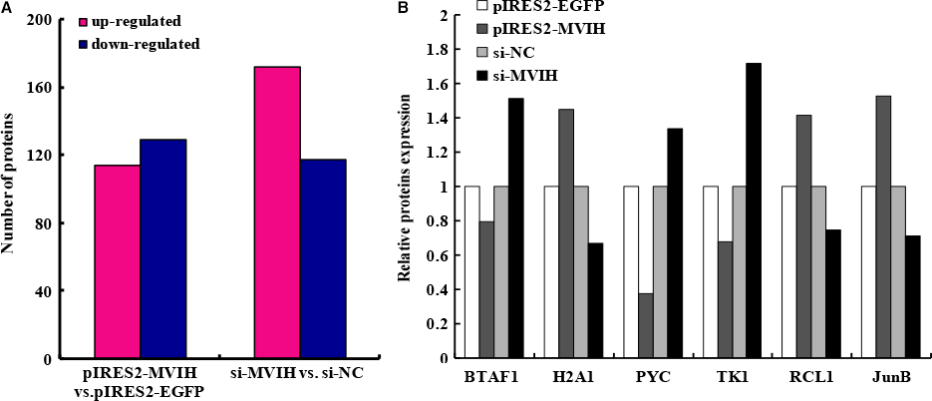
Relevant Protein regulated by *MVIH* (Mass Protein Spectrometric Detection). Performed mass protein spectrometric detection to find relevant proteins which are involved in the role of MVIH in trophoblasts cell lines. A total of 243 proteins were differentially expressed in the group of overexpression of *MVIH* (pIRES2‐MVIH) compared with the control group (pIRES2‐EGFP), in which included 114 up‐regulated and 129 down‐regulated (**A**). Several targets which were associated with cell proliferation or angiogenesis were screened (**B**).

In addition, we determined the expression of vascular endothelial growth factor (VEGF) and its soluble vascular endothelial growth factor receptor‐1 (sFlt‐1), angiopoietin I and II (AngI and AngII) by qPCR after transfection with effective siRNAs *versus* si‐NC and pIRES2‐EGFP *versus* pIRES2‐*MVIH,* respectively. The qPCR analysis revealed that the expression of VEGF, AngI and AngII was down‐regulated to different degrees after *MVIH* knockdown while sFlt‐1 was increased; conversely, the overexpression of *MVIH* promoted the expression of these genes in HTR‐8/SVneo and HUVEC cells (Fig. 6I and J). These results indicate that *MVIH* might modulate the pathogenesis of PE by affecting the formation of vascular endothelial cells and the expression of angiogenesis‐related factors.

### Proteins regulated by *MVIH* as revealed by mass spectrometry

To assess further which proteins were associated with MVIH upon transfection with si‐*MVIH* or pIRES2‐*MVIH*, we then performed mass spectrometry analysis to identify proteins involved in the effects of *MVIH* in the trophoblast cell lines. As shown in Figure 6A, a total of 243 proteins were differentially expressed between the group with *MVIH* overexpression (pIRES2‐*MVIH*) and the control group (pIRES2‐EGFP), among which 114 were up‐regulated and 129 down‐regulated. There were 289 proteins that were differentially expressed after transfection with siRNAs compared with si‐NC in HTR‐8/SVneo cells, of which 172 showed increased and 117 decreased expression.

Data were obtained from clustering analysis performed separately on cells subjected to either down‐regulation or overexpression of MVIH. Six proteins showed a significant difference with contrary tendency between the down‐regulation and overexpression groups, namely, BTAF1 (TATA‐binding protein associated factor 1, Gene ID: 9044), H2A1 (Human Histone H2A type 1, Gene ID: 373337), PYC (Human pyruvate carboxylase in mitochondria, Gene ID: 5091), TK1 (Human Thymidine Kinase, Gene ID: 7083), RCL1 (RNA terminal phosphate cyclase‐like 1, Gene ID: 10171) and JunB (Q6IBG3, Gene ID: 3726) (Fig. 6B). Screening revealed that *JunB* had particular potential to modulate angiogenesis and cell proliferation. Therefore, we focused on *JunB* for further investigation.

### *Jun‐B* is involved in HTR‐8/SVneo cell proliferation, migration and network formation of HUVEC

**Figure 7:**
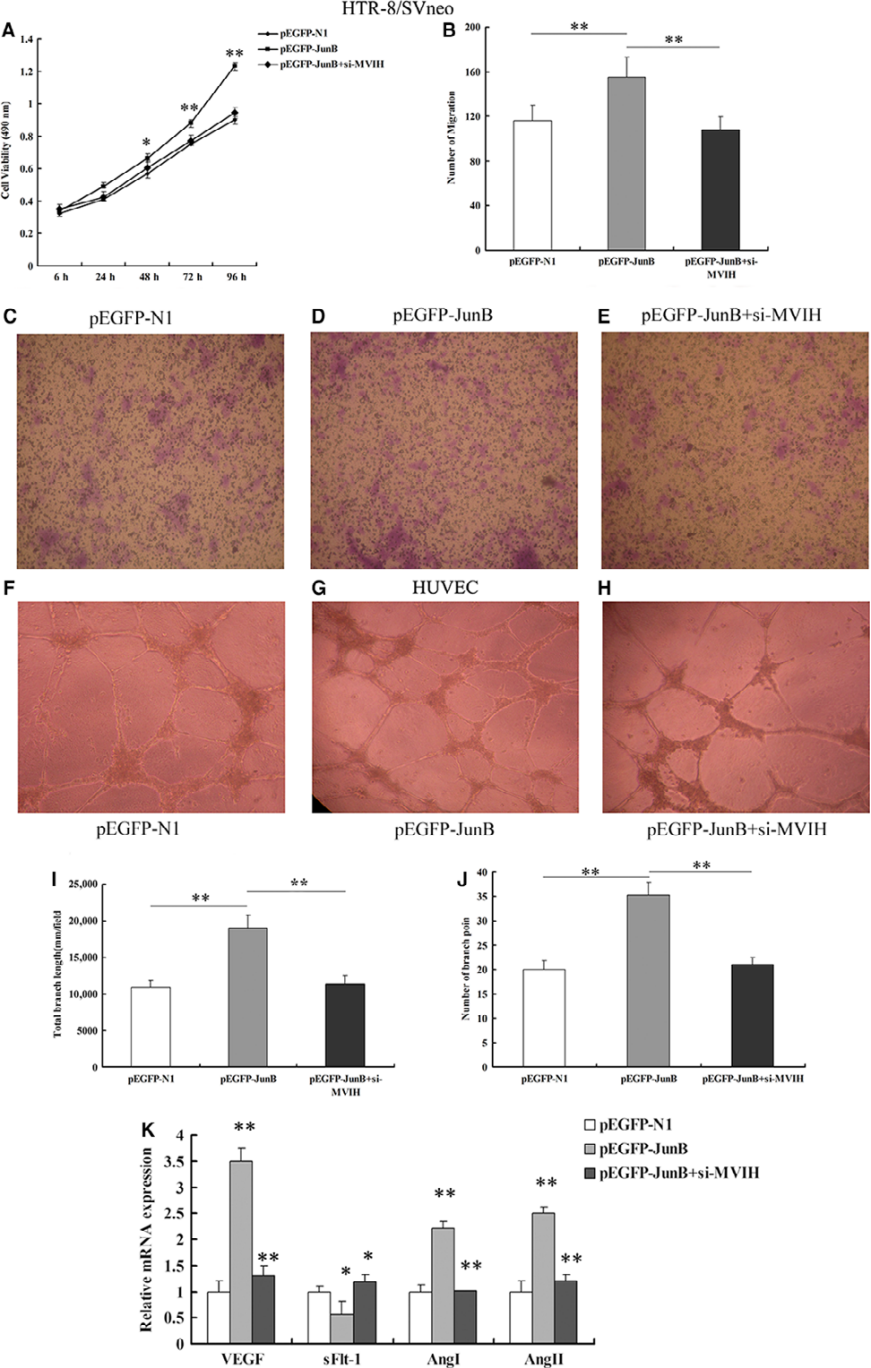
*Jun‐B* involved in cell function phenotype. MTT assays and Transwell assays were used to determine the cell viability and migration for si‐*MVIH* and pEGFP‐Jun‐B cotransfected HTR‐8/SVneo cells, respectively (**A–E**). Network formation assays were performed to examine the network formation ability for si‐*MVIH* and pEGFP‐Jun‐B cotransfected HUVEC cells, respectively (**F–J**). The expression of relevant vascular endothelial growth factor (VEGF) by qPCR after cotransfecting with effective siRNAs and pEGFP‐Jun‐B (**K**). (Values are mean ± SD; *: *P* < 0.05, **: *P* < 0.01)

To confirm the involvement of *Jun‐B* in the biological functions of *MIVH*, we performed cotransfection assays with pEGFP‐*JunB* and si‐*MVIH* in HTR/SVneo cells. MTT assays suggested that the overexpression of *Jun‐B* facilitated the proliferation of HTR‐8/SVneo cells, while the proliferative ability was considerably inferior in the group cotransfected with pEGFP‐*JunB* and si‐*MVIH* (Fig. 7A). The same trend was also found in the migration assays (Fig. 7B–E). The proliferation and migration of *Jun‐B* could be counteracted by silencing *MVIH*, demonstrating that *MVIH* might play a direct regulatory role on *Jun‐B*.

Next, we performed network formation assays to investigate the angiogenesis ability after cotransfection with pEGFP‐*JunB* and si‐*MVIH* in HUVEC cells. The results revealed that stimulation with *Jun‐B* promoted angiogenesis ability; increased the expression of VEGF, AngI and AngII; and decreased the expression of sFlt‐1 compared with the levels in the controls; the opposite trends were revealed after cotransfection with si‐MVIH and *pEGFP‐Jun‐B* (Fig. 7F–K). These findings suggest that *MVIH* and *JunB* have a synergistic effect on the regulation of angiogenesis.

## Discussion

Besides miRNAs, tens of thousands of lncRNAs derived from the genomes of mammals have been identified worldwide 24. Previous studies showed that the dysregulation of lncRNAs may contribute to numerous human diseases in certain organs or tissues 25. The lncRNA family is extremely large, and we still have only an incomplete understanding of the functions and regulatory mechanisms of the lncRNAs identified thus far. Few studies have examined the association between lncRNAs and PE, and these studies mainly focused on aberrant lncRNA expression and its effect on trophoblast cell function. In the present study, we found that the expression of *MVIH* was down‐regulated in the placental tissues of PE compared with that in controls. Previous studies also showed that *MVIH* was up‐regulated in hepatocellular carcinoma tissues and promoted tumour angiogenesis by affecting cell invasion and tube formation ability 19. Based on the main mechanisms impeding uterine spiral artery remodelling in PE 26, we hypothesized that the low expression of *MVIH* is involved in the development of PE. However, it had remained unclear whether *MVIH* regulates the angiogenesis ability of trophoblast cells and vascular endothelial cells in the pathogenesis of PE.

To clarify the biological functions of *MVIH* in PE, we used loss‐ and gain‐of‐function assays to evaluate the ability of *MVIH* to stimulate angiogenesis in HTR‐8/SVneo and HUVEC cells. The results demonstrated that silencing of *MVIH* inhibited the network formation ability of HTR‐8/SVneo and HUVEC cells; the number of capillary‐like networks was considerably decreased, and the expression of vascular endothelial growth factor, angiopoietin I and II was down‐regulated *in vitro*. In contrast, the overexpression of *MVIH* increased the network formation ability and affected the expression of factors associated with angiogenesis correspondingly in trophoblast cells. Therefore, *MVIH* may promote angiogenesis, and low expression of *MVIH* may play an essential role in remodelling of the spiral arteries in PE.

We also performed MTT assays and transwell assays to further establish the various regulatory roles of *MVIH* in different trophoblast cells. The data revealed that knockdown of *MVIH* inhibited cell growth, migration and invasion. However, this knockdown was not proven to be associated with apoptosis. The results showed that the low expression of *MVIH* inhibits the proliferation, migration, invasion and angiogenesis of trophoblast cells, as well as affects the spiral artery remodelling process, which is involved in the pathogenesis of PE.

Given that proteins ultimately affect cell biological processes, we conducted mass spectrometry analysis to determine the expression of proteins regulated by *MVIH*. Considering the results, *JunB* was focused for further investigation for its modulation in angiogenesis and cell proliferation. *Jun‐B,* an important product of the Jun gene family, is a catalytically active endonuclease consisting of 299 amino acids. This protein forms two heterologous polymers with other proteins to form an essential transcription factor, activator protein‐1 27, which regulates cell proliferation and transformation and plays a regulatory role in the occurrence and metastasis of tumours 28. It has been reported that Jun‐B protein stimulates the growth of osteoblasts and chondrocytes by directly activating the transcription of cyclin A 29. In terms of proliferation and angiogenesis, Jun‐B protein can also reverse the proliferation and developmental defects of fibroblast cells induced by c‐Jun deletion 30. In this study, we demonstrated the effect of Jun‐B on the proliferation and migration of trophoblast cells and the ability to undertake endothelial cell tube formation.

In summary, we found that MVIH is down‐regulated in the placental tissues of PE and demonstrated that MVIH exerts vital effects on trophoblast cell invasion and angiogenesis. Taken together, our findings suggest that MVIH is associated with PE and is a candidate biomarker for the diagnosis and treatment of this condition. However, further studies using additional samples and analyses of the biological mechanisms involved are necessary.

## Conflicts of interest

No potential conflicts of interest were disclosed.

## Supporting information


**Table S1** Sequence of primers and siRNAs.

**Table S2** The detailed Mass Spectrometric database and protein identification.
